# Mental distress among adult patients with eosinophilic esophagitis

**DOI:** 10.1111/nmo.14069

**Published:** 2020-12-31

**Authors:** Willemijn E. de Rooij, Floor Bennebroek Evertsz', A. Lei, Albert J. Bredenoord

**Affiliations:** ^1^ Department of Gastroenterology & Hepatology Amsterdam University Medical Center Amsterdam the Netherlands; ^2^ Department of Medical Psychology Amsterdam University Medical Center Amsterdam the Netherlands

**Keywords:** anxiety, depression, Eosinophilic esophagitis, mental care, mental distress

## Abstract

**Rationale:**

Data on the prevalence of mental distress among adult eosinophilic esophagitis (EoE) patients are scarce. Also, a significant gap remains in the understanding of which determinants are related to significant psychological symptoms and whether distressed patients require and receive mental care.

**Methods:**

Adult EoE patients were invited to complete standardized measures on anxiety/depressive symptoms (HADS) and general psychopathology (SCL‐90‐R). All scores were compared to general population norms. Socio‐demographic and clinical factors were assessed.

**Results:**

In total, 147 adult EoE patients (61% males, age 43 (IQR 29–52) years were included (response rate 71%). No difference with general population values was found for total anxiety and depressive symptoms (7.8 ± 6.6 vs. 8.4 ± 6.3; *p* = 0.31). A total of 38/147(26%) patients reported high levels of anxiety and/or depressive symptoms (HADS‐A ≥ 8: 35/147(24%) and HADS‐D ≥ 8: 14/147(10%)), indicative of a possible psychiatric disorder. In a multivariate analysis, age between 18–35 years was independently associated with high levels of anxiety (HADS‐A ≥ 8) (OR 3.0, 95% CI 1.3–6.9; *p* = 0.01). The SCL‐90‐R Global Severity Index (GSI) was significantly higher compared to the general population (*p* < 0.001). Significant signs of general mental distress (GSI ≥ 80th percentile) were observed in 51(36%) EoE patients, of which 29(57%) patients denied having any mental problems and only 8(16%) patients received mental care.

**Conclusion:**

A considerable proportion of adult EoE patients suffers from mental distress, with a 3‐fold risk of significant anxiety in those patients younger than 35 years. Therefore, population‐based studies are required and a proactive approach in the screening for and treatment of these psychological symptoms in EoE practice seems essential.

**Abbreviations**
CI: confidence intervals
EoE: eosinophilic esophagitis
GSI: Global Severity Index
HADS: Hospital Anxiety and Depression Scale
IBD: Inflammatory Bowel Disease
IQR: Interquartile Range
OR: odds ratio
PRO: Patient‐Reported Outcome
SCL‐90‐R: Symptom Checklist‐90‐Revised
SD: Standard Deviation
SDI: Straumann Dysphagia Instrument

**Key Points**

- A significant gap remains in our understanding of the impact on mental health and its determinants in adult EoE patients.
- A considerable proportion of adult EoE patients suffers from mental distress, with a compelling 3‐fold risk of significant anxiety during young adulthood (18–35 years).
- A proactive approach in the screening for and treatment of mental health disorders should therefore become an integral part of the medical care of EoE patients.

## 1 INTRODUCTION

EoE is a chronic immune‐mediated disorder of the esophagus triggered by food allergens, with an Worldwide increasing prevalence with rates almost comparable to inflammatory bowel disease (IBD).^1^, ^2^ EoE is characterized by mucosal eosinophilic infiltration and subsequent esophageal dysfunction, which manifests in symptoms of dysphagia for solid foods and food impaction.^3^ EoE affects all ages (3:1 male‐to‐female ratio), with a peak incidence between the ages of 20 and 40 years.^1^ At present, the management of EoE involves targeting the esophageal eosinophilic inflammation with drugs or elimination of food allergens. EoE is associated with a substantial disease burden that affects patients’ health‐related quality‐of‐life (HRQOL), healthcare systems, and society in general.^4^ Multiple aspects such as disturbing symptoms of dysphagia and food impaction and the need for life‐long treatment are associated with impaired HRQOL.^5^, ^6^, ^7^ A recent medical record review observed a prevalence of psychiatric health comorbidities in almost one‐third of EoE patients, in which older age, female gender, and longer symptom duration were found to be associated with the presence of a mental health disorder.^8^ Current research has mainly focused on increased risk of developing anxiety and depressive symptoms, measured within the construct of disease specific HRQOL (EoE‐QOL‐A).^7^, ^9^, ^10^ This validated measure consists of 5 domains that evaluates important disease‐related topics (e.g., issues related to having a chronic disease or swallowing anxiety) and has been widely used in the EoE‐research field.^4^, ^10^, ^11^ Still a significant gap remains in our understanding of the impact on mental health and its determinants in this chronic disease and if distressed EoE patients receive mental treatment. Notwithstanding, insufficient treatment of psychiatric comorbidities in patients with a chronic physical illness (e.g., IBD and rheumatoid arthritis) has been associated with more severe symptoms and disease flares, therapeutic non‐adherence, and subsequent increased healthcare costs.^12^, ^13^, ^14^ However, provision of sufficient mental care in adult EoE patients first requires more insights into the presence of mental distress and its determinants (e.g., clinical and demographic factors). Therefore, we aimed to evaluate in this study: (a) the presence of mental distress among adult EoE patients, (b) the degree to which clinical and socio‐demographic factors are related to significant levels of mental distress, and (c) if EoE patients with severe symptoms of general mental distress receive mental care.

## 2 METHODS

### 2.1 Study design and population

**FIGURE 1:**
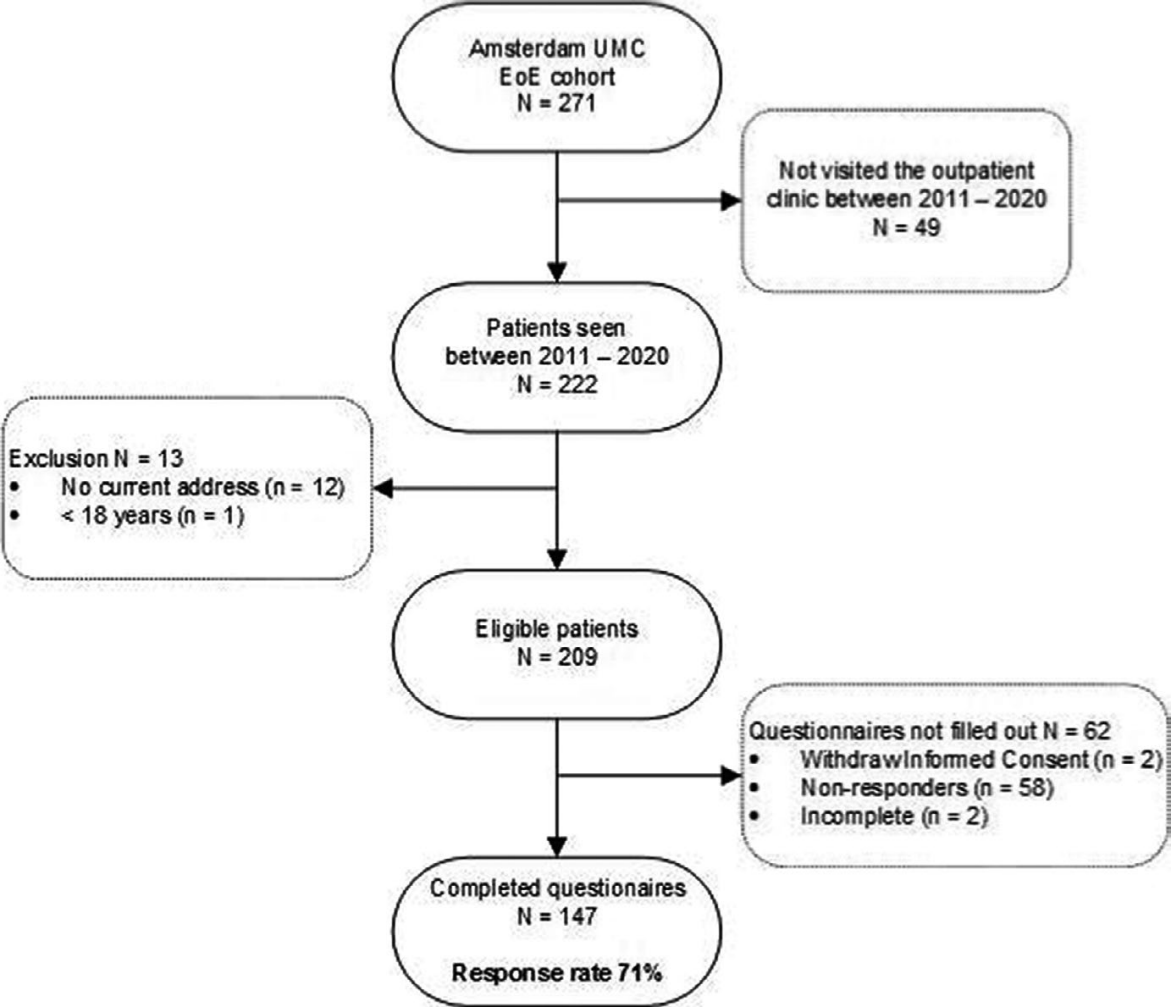
Flowchart of patients inclusion and response rate.

An observational cross‐sectional study design was used to assess mental distress among adult EoE patients. Consecutive patients from our EoE cohort (i.e., patients who attended the outpatient clinical of the Amsterdam UMC Motility Center between 2011 and 2020) were invited to participate in this study between July 2019 and February 2020 (i.e., recruitment period). An informed consent letter including self‐reported questionnaires was sent to the EoE cohort and distributed at the outpatient clinic during this recruitment period. Patients with a documented diagnosis of EoE according to the consensus guidelines (i.e., ≥15 eosinophils per high‐power‐field), aged 18 and over, with a sufficient command of written Dutch to complete a self‐reported survey were considered eligible for inclusion.^3^ Once consented, all patients completed a paper or digital version of the questionnaires. All data were safely collected and stored by using the Electronic Data Capture Castor. A flowchart of patient inclusion and participation rate is presented in Figure 1.

### 2.2 Data collection

#### 2.2.1 Socio‐demographics and clinical outcomes

A self‐designed (standard fixed choice) questionnaire was used to elicit details concerning socio‐demographic and clinical information. Socio‐demographic variables, such as gender and education level (low: primary or secondary school and high: College or University) and specific information on the year of symptom onset and diagnosis of EoE, history of endoscopic interventions and previous dilations, EoE treatment (medical or dietary treatment) and concomitant atopic diseases, were included. In addition, patients were asked if they felt to have current mental health problems and whether they received mental care. Clinical symptoms of dysphagia and food impaction (i.e., clinical disease activity) were evaluated by means of the Straumann Dysphagia Instrument (SDI).^15^ Severe clinical disease activity was defined as current symptoms of daily dysphagia and food impaction.

### 2.3 Study questionnaires and reference population

#### 2.3.1 Anxiety and depression

Anxiety and depressive symptoms were measured with the standardized and validated Hospital Anxiety and Depression Scale (HADS). This 14‐item self‐assessment scale was developed to screen for depression and anxiety symptoms (recall period of 7‐days). The HADS consists of 7 anxiety and 7 depression items, of which the total scores ranges from 0 (no complaints) to 21 (maximum complaints). A score of ≥8 on either subscales signifies a symptom severity indicative for a possible anxiety and/or depressive disorder.^16^ Anxiety and depression symptom scores of all EoE patients were compared to a subgroup of 199 patients, which was derived from 3492 respondents of the general Dutch population.^17^

#### 2.3.2 General mental distress

Symptoms of general mental distress were evaluated by means of the validated Symptom Checklist‐90‐Revised (SCL‐90‐R). This questionnaire consists of 90‐items to assess for general self‐reported psychological symptoms over the past 7 days. The SCL‐90‐R‐items represent 8 domains, including agoraphobia, anxiety, depression, somatization, sensitivity, insufficiency of thinking and acting, hostility and sleep disturbance.^18^ Each item is rated on a 5‐point scale of distress, ranging from 1 (none) to 5 (extreme). The total SCL‐90‐R score (Global Severity Index (GSI)) is calculated by substitution of all subdomain scores and ranges from 0 to 450, with higher scores indicative for mental distress. SCL‐90‐R‐scores of our EoE sample were compared to a reference cohort of 2368 respondents (norm group II) of the Dutch general population.^19^ In addition, cut‐off scores were used to identify patients with severe symptoms of general mental distress, indicated as GSI scores of “above normal” and “high” (corresponding to the 80th percentile of the norm group II), that are clinically relevant and may be indicative of a mental disorder.^19^

### 2.4 Statistical analysis

Statistical analysis was performed by using IBM SPSS Statistics (version 25.0) (SPSS, Chicago, USA). Descriptive statistics was used to assess socio‐demographic and clinical characteristics. Data are presented as mean (±Standard Deviation (SD)) or median (interquartile range (IQR)). To characterize our sample, levels of the validated Patient‐Reported Outcome (PRO) measures (HADS/SCL‐90‐R) were compared to previously published general population norms.^17^, ^18^ Independent sample t tests were used to compare mean scores of the HADS and SCL‐90‐R in EoE patients to the general population norms. Univariate logistic regression analyses were performed to identify (clinical relevant) factors associated with high levels of anxiety (HADS‐A ≥ 8). Demographic variables with a *p*‐value of <0.20 were subsequently entered into multivariate logistic regression analysis with backward selection. Associations between clinical disease activity (SDI scores) and HADS‐A and HADS‐D and all subscales of the SCL‐90‐R were assessed by Pearson's or Spearman's rank correlations coefficients, as appropriate. A *p*‐value of <0.05 was considered to be statistical significant.

### 2.5 Ethical considerations

This cross‐sectional study was conducted according to the principles of the Declaration of Helsinki and in accordance with the Medical Research Involving Human Subjects Act (WMO). An exemption to seek formal approval was provided by the Medical Ethics Committee of the Amsterdam UMC at 25‐03‐2019 (W19_103#19.136). All participants provided informed consent before taking part and were given an unique study‐ID to ensure anonymity.

## 3 RESULTS

### 3.1 Patient characteristics

In total, 147 adult EoE patients were included (61% males, median age 43 (IQR 29–52) years), representing a response rate of 71%. Atopic constitution was observed in 119 (81%) patients. The median disease duration in our cohort was 3 (IQR 1–6) years, with 49 (33%) patients diagnosed within the prior year. Diagnostic delay, measured as time interval between first reported EoE symptoms and year of diagnosis, was 5 (IQR 2–14) years. In total, 21 (14%) patients had prior esophageal dilation and multiple endoscopic interventions with food bolus extraction were reported in 62 (42%) patients (Table 1).

**TABLE 1:** Socio‐demographic and clinical characteristics.

	EoE, *n* (%) or median (IQR) (*n* = 147)
Socio‐demographic characteristics	
Age, *years*	43 (29–52)
Gender, *male*	90 (61)
Level of education	
Low	49 (33)
High	98 (67)
In domestic partnership	
No	51 (35)
Yes	96 (65)
Clinical characteristics	
Atopic diatheses	119 (81)
Current clinical disease activity	
Dysphagia	97 (66)
Food impaction	41 (28)
Multiple endoscopic interventions with food bolus extraction	62 (42)
Diagnostic delay^a^, *years*	5 (2–14)
Disease duration, measured from year of diagnosis, *years*	3 (1–6)
Age at symptom onset, *years*	27 (19–38)
Previous dilation	21 (14)
Current treatment	
Topical steroids	35 (24)
Dietary restrictions	35 (24)
Topical steroids with additional dietary restrictions	15 (10)
PPIs	34 (23)
No treatment	28 (19)

IQR, Interquartile Range; PPIs, Proton‐Pump Inhibitors.

^a^ Diagnostic delay is the time interval between the first symptoms and the diagnosis.

### 3.2 Anxiety and depressive symptoms

**FIGURE 2:**
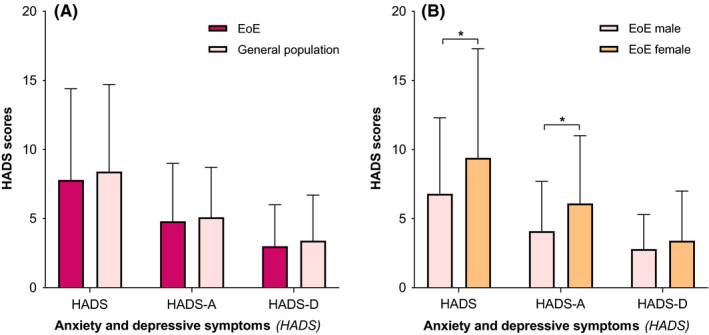
**(A)** Anxiety and depressive symptoms (HADS) of eosinophilic esophagitis (EoE) patients vs. the general population. HADS, Hospital Anxiety and Depression Scale, HADS‐D, HADS Depression, HADS‐A, HADS Anxiety *p‐value of <0.05, indicating a significant outcome. **(B)** Anxiety and depressive symptoms (HADS) of male vs. female eosinophilic esophagitis (EoE) patients. HADS, Hospital Anxiety and Depression Scale, HADS‐D, HADS Depression, HADS‐A, HADS Anxiety *p‐value of <0.05, indicating a significant outcome.

Evaluation of anxiety and/or depressive symptoms (HADS) showed no difference in the total HADS score in our EoE sample compared to the general population (7.8 ± 6.6 vs. 8.4 ± 6.3; *p* = 0.31) (Figure 2). Anxiety (HADS‐A) and depression (HADS‐D) symptom scores in EoE patients were also both similar compared to the general population (HADS‐A: 4.8 ± 4.2 vs. 5.1 ± 3.6; *p* = 0.47 and HADS‐D: 3 ± 3 vs. 3.4 ± 3.3; *p* = 0.1), respectively (Figure 2A). Additionally, no differences were observed for the HADS‐total, HADS‐A and HADS‐D mean scores in female EoE patients compared to the general population (all; *p* > 0.05). Moreover, male EoE patients showed significantly lower HADS‐total, HADS‐A, and HADS‐D scores compared to the general population (all; *p* < 0.05). In our EoE sample, significantly higher levels of the HADS‐total score in females were observed compared to males (9.4 ± 7.9 vs. 6.8 ± 5.5; *p* = 0.02) (Figure 2B). Furthermore, significant higher levels of the HADS‐A were detected in female EoE patients compared to males (6.1 ± 4.9 vs. 4.1 ± 3.6; *p* = 0.005), whereas HADS‐D scores between male and female patients were similar (2.8 ± 2.5 vs. 3.4 ± 3.6; *p* = 0.226) (Figure 2B).

In our cohort, high levels of anxiety (HADS‐A ≥ 8; indicative of an anxiety disorder) were observed in 35 (24%) patients, with no gender difference (male vs. female; *p* = 0.11). High levels of depression (HADS‐D ≥ 8; indicative of an depressive disorder) were reported in 14 (10%) patients, whereas females were significantly more affected compared to males (6% vs.16%; *p* = 0.048). Furthermore, 14 (10%) patients had high levels of both anxiety and depression (HADS‐A ≥ 8 and HADS‐D ≥ 8; indicative of both psychiatric disorders), of which the proportion of females was significantly higher (male 3% vs. female 14%; *p* = 0.023). Hence, a total of 38 (26%) patients (no difference between male vs. female; *p* = 0.123) scored high levels of anxiety and/or depressive symptoms; indicative of at least one of these psychiatric disorders.

### 3.3 Associated factors with high levels of anxiety

Presence of high levels of anxiety (HADS‐A ≥ 8; indicative of an anxiety disorder) was significantly more prevalent in young patients aged between 18–35 years (41%). Univariate analysis signified a possible trend between high levels of anxiety and younger age (18–35 years), female gender, not being in domestic partnership, current symptoms of daily dysphagia and food impaction (severe clinical disease activity) and a short disease duration (≤2 years). However, after multivariate logistic regression analysis, age between 18–35 years was the only independent factor associated with high levels of anxiety (odds ratio (OR) 3.0, 95% confidence interval (CI) 1.3–6.9; *p* = 0.01 (Table 2).

**TABLE 2:** Determinant factors associated with high levels of anxiety.

EoE patients *N* = 147	HADS‐A ≥ 8 *n* (%)	Univariate analysis					Multivariate analysis			
High levels of anxiety *N* = 35		OR	CI (95%)		*p*‐value		OR	CI (95%)		*p*‐value
Demographic variables										
Female gender	18 (32)	1.982		0.919 to 4.274		0.081†	NS			NS
Age										
18–35	21 (41)	3.123		1.375 to 7.092		0.007^‡^	2.999		1.307 to 6.881	0.01^‡^
36–55	13 (18)	Ref.								
>55	1/25 (4)									
In domestic partnership	19 (20)	0.540		0.248 to 1.173		0.119†				NS
Severe clinical disease activity	6 (46)	3.143		0.763 to 12.945		0.113†				NS
Short disease duration (≤2 years)	19 (31)	1.906		0.886 to 4.100		0.099^‡^				NS

Disease duration, measured from year of diagnosis.

OR, Odds Ratio; CI (95%), 95% Confidence interval; NS, Not significant; EoE, Eosinophilic esophagitis; P, percentile.

Severe clinical disease activity =currently experiencing symptoms of daily dysphagia with food impaction

^†^ *p*‐value <0.2, indicating a possible trend.

^‡^ *p*‐value of <0.05, indicating a significant outcome.

### 3.4 General mental distress

**FIGURE 3:**
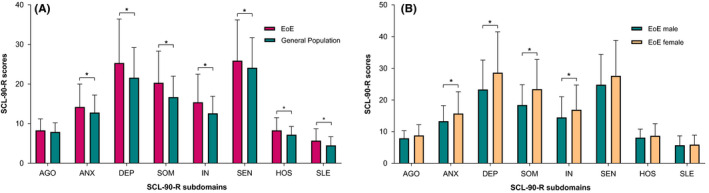
**(A)** Mean scores on the subscales of the Symptom Checklist 90–Revised (SCL‐90‐R) of patients with eosinophilic esophagitis (EoE) vs. the general population. AGO, Agoraphobia; ANX, Anxiety; DEP, Depression; SOM, Somatic Symptoms; IN, Inadequacy of Thinking and Acting; SEN, Distrust and Interpersonal Sensitivity; HOS, Hostility; and SLE, Sleeping * P‐value of <0.05, indicating a significant outcome.**(B)** Mean scores on the subscales of the Symptom Checklist 90–Revised (SCL‐90‐R) of male vs. female patients with eosinophilic esophagitis (EoE). AGO, Agoraphobia; ANX, Anxiety; DEP, Depression; SOM, Somatic Symptoms; IN, Inadequacy of Thinking and Acting; SEN, Distrust and Interpersonal Sensitivity; HOS, Hostility; and SLE, Sleeping *p‐value of <0.05, indicating a significant outcome.

The general psychopathological profile of EoE patients was evaluated by means of self‐reported symptoms of general mental distress (SCL‐90‐R), showing significantly higher levels of the GSI compared to the general population (135 ± 47.3 vs. 118.3 ± 32.3; *p* < 0.001). In addition, levels of the symptom subscales; depression, somatization, insufficiency of thinking and acting, hostility and sleep disturbance and anxiety and sensitivity in EoE patients were all significantly higher compared to the general population (*p* < 0.001 and *p* < 0.05), respectively (Figure 3A). In addition, GSI levels of both male and female EoE patients were significantly higher compared to the general population (males: 131.7 ± 44.9 vs. 118.3 ± 32.3; *p* = 0.005 and females: 140.5 ± 51 vs. 118.3 ± 32.3; *p* = 0.002), respectively. The subscales; anxiety, depression, somatization and insufficiency of thinking and acting were significantly higher in both male and female EoE patients compared to the general population (all; *p* < 0.05). In our EoE cohort, female patients showed significantly higher levels of the GSI compared to male patients (127.3 ± 42 vs. 147.8 ± 52; *p* = 0.017) and the subscales anxiety, depression, somatization and insufficiency of thinking and acting (male vs. female; *p* < 0.05) (Figure 3B).

**FIGURE 4:**
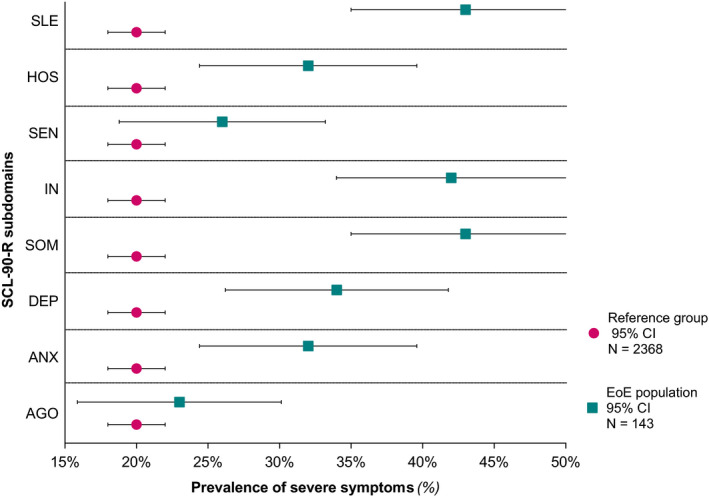
Presence of severe symptoms on the subscales of the Symptom Checklist 90–Revised (SCL‐90‐R) in patients with eosinophilic esophagitis (EoE). Each dimension presents the percentage of EoE patients exceeding the norm scores indicated as “above normal” and “high” (≥80th percentile norm group II).^16^ AGO, Agoraphobia; ANX, Anxiety; DEP, Depression; SOM, Somatic Symptoms; IN, Inadequacy of Thinking and Acting; SEN, Distrust and Interpersonal Sensitivity; HOS, Hostility; and SLE, Sleeping.

Severe symptoms of general mental distress, indicated as GSI scores of “above normal” and “high” (corresponding to the 80^th^ percentile of the norm group II), were observed in 51 (36%) EoE patients, of which the proportion of females was significantly higher than males (46% vs. 29%; *p* = 0.048). Evaluation of the symptom subscales for general mental distress (SCL‐90‐R) showed a significantly higher proportion of females with severe symptoms of depression (SCL‐90‐depression ≥80th percentile) and somatization (SCL‐90‐somatization ≥80th percentile) (male vs. female; *p* = 0.029 and *p* = 0.001), respectively. The percentages of patients in our EoE population exceeding the norm scores indicated as “above normal” and “high” (≥80^th^percentile) in all dimensions of the SCL‐90‐R are presented in Figure 4.

In total, 22 (43%) patients with severe symptoms of general mental distress (GSI ≥80^th^percentile) reported to have current mental problems, of which only 8 (36%) patients received mental care and psychotropic medication (e.g., antidepressants or anxiolytics) was used in 7 (14%) patients. Fifteen (29%) patients with severe symptoms of general mental distress felt their mental problems were related to EoE. Of note, 29 (57%) patients with GSI scores exceeding the norm scores (≥80th percentile) denied having any mental problems.

### 3.5 Associations between clinical disease activity and symptoms of mental distress

Ninety‐seven (66%) patients reported current symptoms of dysphagia and 41 (28%) food impaction, of which 33 (81%) stated to have multiple episodes a week. Comparison of self‐reported clinical disease severity and HADS‐scores showed a significant positive correlation between the total SDI scores and both the HADS‐A (*r* = 0.27; *p* = 0.001) and HADS‐D (*r* = 0.19; *p* = 0.023) scores (Table S1). Additionally, SCL‐90‐R‐subscales agoraphobia, anxiety, depression, somatization, sensitivity, insufficiency of thinking and acting, hostility and sleep disturbance all showed a significant positive correlation with the total SDI score (all; *p* < 0.05). (Table S1).

## 4 DISCUSSION

EoE is known to have impact on HRQOL of patients who suffer from the disease, although current literature is scarce on the understanding of mental health comorbidities in adult EoE patients. In this cross‐sectional study, we observed a substantial presence of significant symptoms of mental distress among adult EoE patients. Although mean levels of anxiety and depression in our sample were not higher compared to the general population, relevant signs of anxiety (HADS‐A ≥ 8; indicative of an anxiety disorder) were seen in 24% patients. Moreover, high levels of depression (HADS‐D ≥ 8) were noted in 14 (10%) patients. These observed rates are comparable to a study of Lucendo et al, in adult EoE patients, reporting significant signs of anxiety and depression in 31% and 10%, respectively.^9^ Furthermore, a remarkable finding in our study was the significant 3‐fold risk for the presence of high levels of anxiety (HADS‐A ≥ 8) in EoE patients between the ages of 18 and 35 years. The general onset of anxiety disorders usually occurs in childhood/adolescence, until they reach a peak in middle age, with tendency to decrease with older age.^20^ With regard to EoE, a pediatric study suggested anxiety symptoms to increase with age, including rates of 9.3% in children (<11 years) and 19% in adolescents (11–17 years).^21^ In our EoE sample, 41% of young adults (18–35 years) and 18% of the middle‐aged (36–55 years) patients presented with significant signs of anxiety. Compared to prevalence rates of anxiety (HADS‐A ≥ 8) in a general German population, which ranges from 14.4%–19.8% (<40 years) and 19.8%–25% (41–60 years),^22^ it is certain that young adults diagnosed with EoE are more at risk for the development of significant signs of anxiety.

Overall, females showed significantly higher levels of mental distress compared to males in our EoE sample. This finding is consistent with previous literature reports on female predominance of common mental disorders in the general population.^23^, ^24^ For that reason, it seems notable that the proportion of males and females with significant signs of anxiety on both PRO measures (HADS‐A ≥ 8 and SCL‐90‐anxiety ≥80^th^ percentile) were equally distributed in our EoE sample. Since men are more prone of stricture development with consecutive risk of increased symptom severity,^25^ one could argue that male EoE patients are more exposed to potential anxiety triggers such as impaction with need for upper endoscopy and food bolus dislodgement. This is supported by previous findings on the serious impact of dysphagia and food impaction on patients’ fear, and identification of increased symptom severity as predictor of both disease and chocking anxiety.^7^, ^10^ Although severe clinical disease activity was not independently associated with high levels of anxiety in our multivariate analysis, SDI scores significantly correlated with scores of the HADS‐A and SCL‐90‐anxiety (Table S1).

Compared to the general population, a greater severity of mental distress in EoE patients was observed, with a substantial proportion of patients (36%) with severe symptom levels (GSI ≥80^th^ percentile) in our sample. Nevertheless, these results should be interpreted with caution, since the SCL‐90‐R is not corrected for somatic disorders.^18^ In addition, the HADS anxiety and depression scores were not higher compared to the general population, whereas the SCL‐90‐R‐subscales anxiety and depression were significantly higher in EoE patients. Although a clear explanation is lacking, this inequality might be the result of the HADS being corrected for the presence of physical illness.^17^ Also, a more extensive screening as result of a higher number of items included in the SCL‐90‐R, in particular in the domain depression, might also be suggested as an explanation for this contrasting finding. Moreover, considering somatization (i.e., SCL‐90‐somatization) to be the most intense symptom in our sample, it could be argued that the presence of physical illness resulted in an overestimation of the GSI score (Figure 3A and Figure 4). However, only the questions “pain in the chest or heart” (item 12) and “having a lump in the throat” (item 53) fits with EoE‐related symptoms, suggesting these patients actually may experience somatic symptoms (e.g., difficulty to breath or dizziness) in response to their psychological distress. Moreover, the presence of EoE‐related symptoms (SDI scores) significantly correlated with SCL‐90‐somatization levels (*r* = 0.4; *p* < 0.001), even if corrected for EoE‐related symptoms by exclusion of SCL‐90‐items 12 and 53 (Table S1). Generally, there is a moderate association between symptoms and biological disease activity (esophageal inflammation) in non‐dilated EoE patients.^26^, ^27^

We hypothesize that somatization of esophageal symptoms (e.g., dysphagia) in severe distressed EoE patients may help to explain additional variation in symptom severity, once variation in biological disease activity has already been taken into consideration. In IBD‐patients, association between somatization and clinically active disease with absence of mucosal inflammation, was suggested to be secondary to somatoform‐type behavior or a coexisting functional disease instead of being related to biological disease activity (i.e., mucosal inflammation or extraintestinal manifestations of IBD).^28^ The concept of this so‐called somatoform‐type behavior might also play a role in EoE; the absence of histological data in our cohort did not allow us to further address this hypothesis.

Despite their clinical and public health importance, the presence of psychological disorders is often underdiagnosed and undertreated, in particular when coexisting with physical illness.^29^ Significant signs of general mental distress (GSI ≥80^th^ percentile) were observed in 51 (36%) EoE patients, of which 29 (57%) patients denied having any mental problems. Also, only 8 (16%) of these patients received mental care of which 7 (14%) patients reported current psychotropic medication use. Therefore, routine screening by gastroenterologists for symptoms of anxiety and depression in adult EoE patients through the mental health subscale of the Short Form (SF)‐36 or Patient Health Questionnaire (PHQ)‐4 could be suggested for clinical practice.^30^, ^31^, ^32^ As such, several drivers of disease‐related anxiety, such as symptom severity and need for long‐term food restrictions, have been indicated to be legitimate concerns for care givers in pediatric EoE. Significant impacts on eating and food‐specific anxieties emerging into a newly classified eating disorder; Avoidant/Restrictive Food Intake Disorder (ARFID) has already been observed in pediatric EoE and other digestive diseases.^33^, ^34^ ARFID is characterized by extreme restrictive eating behaviors (i.e., disturbed feeding patterns, highly selective eating habits) and awareness on the presence of this specific mental disorder in adult EoE patients should also be increased.

Based on our results, it remains unclear whether distressed EoE patients’ felt they received the mental care they need. The World Health Organization (WHO) studied the consultation process for mental health reasons, in which the preference for self‐management (i.e., managing one's self) has been indicated as main barrier for not seeking mental treatment, even though need for mental care was perceived.^35^, ^36^, ^37^ In addition, especially young‐ and middle‐aged patients are more likely to recognize need for treatment but experience more structural barriers to treatment seeking, such as negative attitude toward help seeking, financial problems, and time barriers.^38^ Therefore, also a proactive approach toward (unmet) needs for mental care could be suggested for clinical practice.

Several limitations of our study merit attention. First, including patients from a tertiary center is known for limiting the generalizability of outcomes. However, as we included patients from our EoE cohort and new patients visiting the outpatient clinic, our study sample reflects a various population containing different stages of disease activity. Additionally, considering patients with mental disorders often face stigma, psychotropic medication use may have been underreported in our study. Nevertheless, these limitations are encountered by several strengths of our study design. To the best of our knowledge, this is the first cross‐sectional study with specific interest of evaluating the presence of mental distress among adult EoE patients and the extent to which clinical and socio‐demographic factors are related. Considering the use of 2 validated PRO measures (HADS/SCL‐90‐R), new insights are provided on the psychopathological profile of adult EoE patients. Another strength of our study lies in the large sample size of our cohort including EoE patients from various geographical areas in the Netherlands.

In conclusion, we observed a substantial presence of mental distress among adult EoE patients, with a compelling 3‐fold risk of significant signs of anxiety during young adulthood (18–35 years). These findings are highlighting the need for future population‐based studies on the prevalence of mental distress. Since EoE mostly affects young adults, screening for and treatment of mental health disorders should therefore become an integral part of the medical care of EoE patients.

## Supporting information

Table S1‐2
