# Supplementary material for: Mental distress among adult patients with eosinophilic esophagitis
Source: Neurogastroenterol Motil. 2020 Dec 31;33(7):e14069. doi: 10.1111/nmo.14069 (PMC8365712; doi:10.1111/nmo.14069)
Supplement: Supplementary file 1 — Table S1‐2 [file NMO-33-e14069-s001.docx]

| **Supplementary Table 1. \|** Correlations between clinical disease activity and HADS and SCL-90-R in adult EoE patients | | | |
| --- | --- | --- | --- |
|  | **Clinical disease activity (SDI-score)** | | |
| **Symptoms of mental distress** | | **r** | **P value^a^** |
| **HADS** | |  |  |
| HADS-A | | 0.27 | 0.001 ** |
| HADS-D | | 0.19 | 0.023 * |
|  | |  |  |
| **SCL-90-R** | |  |  |
| Agoraphobia | | 0.17 | 0.042 * |
| Anxiety | | 0.3 | < 0.001 *** |
| Depression | | 0.34 | < 0.001 *** |
| Somatization | | 0.44 | < 0.001 *** |
| Somatization (corrected) ^i^ | | 0.4 | < 0.001 *** |
| Sensitivity | | 0.29 | < 0.001 *** |
| Insufficiency of thinking and acting | | 0.34 | < 0.001 *** |
| Hostility | | 0.27 | 0.001 ** |
| Sleep distubance | | 0.26 | 0.002 ** |
| EoE = Eosinophilic esophagitis, HADS = Hospital Anxiety and Depression Scale, HADS-D = HADS Depression, HADS-A = HADS Anxiety, SCL-90-R = Symptom Checklist (90) Revised, SDI-score = Straumann Dysphagia Instrument score  ^I^ SCL-90-R items 12 and 53 that may be related to EoE symptoms are excluded  ^a^ P value correlations between the total SDI-scores and HADS or SCL-90-R domains (Pearson’s or Spearman’s Rank correlation coefficients, as appropriate)  * P value of < 0.05, indicating a significant outcome  ** P value of < 0.01  *** P value < 0.001 | | | |
